# Cross‐Reactive IgG Antibody Responses to SARS‐CoV‐2 in Older Adults Following Seasonal Coronavirus Infection in Jiangsu, China (2015–2017)

**DOI:** 10.1111/irv.70250

**Published:** 2026-03-13

**Authors:** Cheng Xiao, Nancy H. L. Leung, Shiman Ling, Xia Lin, Yuyun Chen, Yanmin Xie, Malik Peiris, Mark Zanin, Benjamin Cowling, Sook‐San Wong

**Affiliations:** ^1^ HKU‐Pasteur Research Pole, School of Public Health, Li Ka Shing Faculty of Medicine The University of Hong Kong Hong Kong Special Administrative Region China; ^2^ WHO Collaborating Centre for Infectious Disease Epidemiology and Control, School of Public Health, Li Ka Shing Faculty of Medicine The University of Hong Kong Hong Kong Special Administrative Region China; ^3^ State Key Laboratory for Respiratory Diseases Guangzhou Medical University Guangzhou China; ^4^ School of Public Health, Li Ka Shing Faculty of Medicine The University of Hong Kong Hong Kong Special Administrative Region China; ^5^ Center for Infection and Immunology Hong Kong Science Park Hong Kong Special Administrative Region China

## Abstract

The extent of cross‐reactive antibodies to SARS‐CoV‐2 elicited by seasonal human coronaviruses (HCoVs) remains unclear. We analyzed longitudinal preinfection and postinfection IgG responses in 62 older adults with PCR‐confirmed HCoV infections from sera collected prior to the emergence of SARS‐CoV‐2. At baseline, 12.9% and 16.1% had low‐titer antibodies against SARS‐CoV‐2 spike (S) or nucleocapsid (N) proteins, respectively, but postinfection increases were marginal. Our findings suggest seasonal HCoV infections induce limited SARS‐CoV‐2 cross‐reactive antibodies in community‐dwelling older adults.

## 1 Introduction

The emergence of SARS‐CoV‐2 has raised significant interest about potential antibody cross‐reactivity with seasonal human coronaviruses (HCoVs) HCoV‐OC43, HCoV‐HKU1, HCoV‐229E, and HCoV‐NL63, which share approximately 60% genetic sequence similarity with SARS‐CoV‐2 [1]. Cross‐reactive antibodies to SARS‐CoV‐2 have been identified in prepandemic serum samples, particularly against the more conserved viral proteins. For example, the frequency of SARS‐CoV‐2 S2 antibodies was highest in children up to 16 years old that were unexposed to SARS‐CoV‐2 [2]. Further, pre‐existing seasonal coronavirus antibodies can negatively impact subsequent de novo antibody responses against SARS‐CoV‐2 [3].

Despite extensive discussion on cross‐reactive antibodies and memory B‐ and T‐cell responses between HCoVs and SARS‐CoV‐2, the impact of this immunological cross‐reactivity remains unclear. Sagar and colleagues found that although a recent HCoV infection did not prevent SARS‐CoV‐2 infection, it was associated with reduced COVID‐19 disease severity, suggesting potential cross‐protective mechanisms mediated by pre‐existing HCoV immunity [4]. This pre‐existing immunity may preferentially boost responses to conserved epitopes shared by HCoVs and SARS‐CoV‐2, consistent with “original antigenic sin” [5]. McNaughton et al. reported that severe COVID‐19 cases were associated with a lower de novo antibody response to the Receptor‐Binding‐Domain (RBD) and N‐Terminal Domain (NTD) of the SARS‐CoV‐2 spike (S) protein, compared with the more conserved epitopes [5]. These may impair de novo antibody production against novel SARS‐CoV‐2 epitopes.

Current investigations of this pre‐existing immunity largely depend on the hypothesis that prepandemic populations had been exposed to HCoVs, with cross‐reactivity presumed to originate from prior HCoV infections [6]. However, rates of HCoV cases and seropositivity could vary substantially by age groups and geographic region [7]. It is important to determine if, and to what extent, HCoV immunity contributes to SARS‐CoV‐2 cross‐reactive immunity. Here, we studied the cross‐reactive IgG antibody profiles before and after PCR‐confirmed seasonal coronavirus infections in a well‐defined cohort of older adults from southeastern China (2015–2017) [8]. This longitudinal design provides direct evidence of infection‐induced antibody responses. Our findings provide insights into the nature and extent of antibody cross‐reactivity between seasonal HCoVs and SARS‐CoV‐2.

## 2 Methods

Samples were collected from the China Ageing REespiratory infections Study (CARES) cohort completed before the COVID‐19 pandemic. The detailed description and protocol [8] as well as the main study findings [9] were available elsewhere. In brief, this prepandemic cohort spanned from December 2015 to September 2017 and comprised older adults aged 60–89 years at enrolment in Jiangsu Province, China. Blood samples were collected every 6 months from enrolment. Year‐round active surveillance with weekly phone calls to identify acute respiratory illness (ARI) was conducted, and mid‐turbinate nasal and oropharyngeal swabs were collected during eligible ARI episodes identified through active surveillance for confirmation of infection by PCR [9].

We screened 844 swabs from 492 participants collected during their active ARI for four human coronaviruses: the alphacoronaviruses HCoV‐229E and HCoV‐NL63, and the betacoronaviruses HCoV‐OC43 and HCoV‐HKU1. Screening was performed using quantitative reverse transcription polymerase chain reaction (RT‐qPCR), as previously described [10]. We identified 62 HCoV PCR‐positive cases where the longitudinal sera at baseline (pre‐infection) plus at least one follow‐up timepoint (post‐infection) were available and then determined the anti‐full‐length S (S1 + S2), anti‐S S1 subunit, antinucleocapsid (N) IgG, and the potential cross‐reactivity to SARS‐CoV‐2 (a betacoronavirus) antigens by enzyme‐linked immunosorbent assay (ELISA). Endpoint titers were defined as the highest reciprocal dilution with a positive/negative signal ratio ≥ 2. Titers ≥ 100 were defined as seropositive.

The following serum timepoints were analyzed: baseline Timepoint 0 (T0, median of 102 days before infection, IQR: 73–147, *n* = 62) and maximum four follow‐up timepoints after infection, where Timepoint 0 was defined as the most recent serum collected right before the HCoV infection, and Timepoints 1–4 were defined as the *n*th serum collected post‐infection: Timepoint 1 (T1, median 95 days post infection; IQR: 48–133, *n* = 62), Timepoint 2 (T2, median: 263 days post infection; IQR: 200–296, *n* = 47), Timepoint 3 (T3, median: 426 days post infection; IQR: 384–460, *n* = 30), and Timepoint 4 (T4, median: 595.5 days post infection; IQR: 547.5–612.8, *n* = 6). The number of postinfection samples available per participant depended on their infection timing relative to the end of the CARES study. Participants with later infections only contributed to the initial postinfection timepoints (e.g., T1, T2) before blood collection ended.

A control group (*n* = 10) was selected from participants who reported ARI but tested PCR‐negative for all four HCoVs. Controls were matched to the cases based on age, sampling city (4 from Yancheng, 6 from Suzhou). Serum samples from baseline to postinfection timepoints up to T3 were analyzed for these controls.

## 3 Results

**FIGURE 1:**
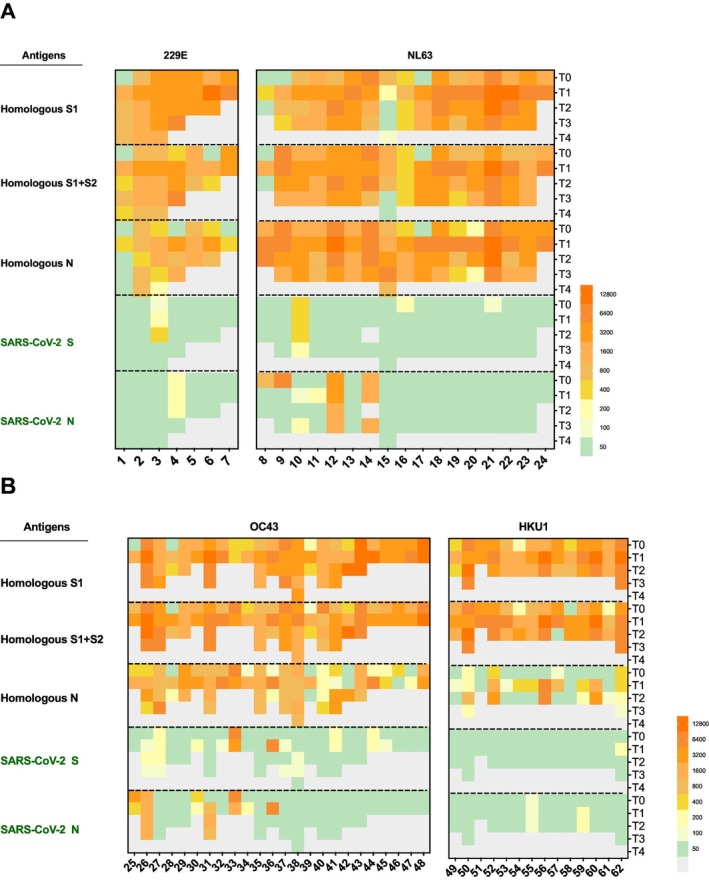
IgG antibody profile of HCoV‐positive individuals (*N* = 62). Cross‐reactive antibody titers to SARS‐CoV‐2 were limitedly increased after HCoV infection. Individual IgG titers against the homologous and SARS‐CoV‐2 proteins in (A) alphacoronaviruses HCoV‐229E (*N* = 7) and HCoV‐NL63 cases (*N* = 17), and (B) betacoronaviruses HCoV‐OC43 (*N* = 24) and HCoV‐HKU1 cases (*N* = 14) were shown in heatmap. Each panel included participants infected by one particular HCoV and showed IgG responses to antigens of that particular HCoV as well as to that of SARS‐CoV‐2. T0 indicates preinfection timepoint and T1–T4 indicate follow‐up timepoints. Titer below detection threshold were shown in green color and absent samples were shown in gray color.

Longitudinal preinfection and postinfection sera from a total of 62 participants with PCR‐confirmed HCoV infections were analyzed (Table 1). Eight baseline sera were seropositive to SARS‐CoV‐2 S protein (12.9%), with relatively low endpoint‐titers (100–400) detected in all except one in the HCoV‐OC43 infected group (participant 33). Ten (16.1%) were seropositive to SARS‐CoV‐2 N protein, with five individuals in the HCoV‐NL63 and HCoV‐OC43 groups showing persistent high titers (> 800) at more than two time points (Figure 1), indicating that the pre‐existing antibodies to SARS‐CoV‐2 in most of the individuals were not transient. Interestingly, baseline cross‐reactive seropositivity to SARS‐CoV‐2 S or N antigens were mutually exclusive and only one individual showed both SARS‐CoV‐2 S and N antibody titers (participant 33, Figure 1B).

**TABLE 1:** Demographics and infection status of participants.

Category	*N*	%
Female	35	56.5
Male	27	43.5
HCoV‐229E	7	11
HCoV‐NL63	17	27
HCoV‐OC43	24	39
HCoV‐HKU1	14	23
	**Median**	**Interquartile range**
Age (years)	74	67–80

By T1, OC43‐infected cases had the most cross‐reactive antibodies against SARS‐CoV‐2. 25% (6/24) of cases showed increased anti‐S IgG from GMT of 73 (95% CI: 46–114) to 92 (95% CI: 60–177) after infection (Table 2); 12.5% (3/24) showed increased anti‐N IgG level from GMT of 92 (95% CI: 50–169) to 112 (95% CI: 61–206). Seroconversion here is defined as a conversion from undetectable antigen‐specific antibody level (below the threshold of 100) to a seropositive status after infection (with titer of 400 or above), or a fourfold or greater increase in antibody titers in those with pre‐existing antibodies. The seroconversion of cross‐reactive antibodies was only observed during HCoV‐OC43 infection—one case seroconverted to SARS‐CoV‐2 S (Participant No. 31) and one case to both S and N protein (Participant No. 36). There were no statistically significant differences in the magnitude of antibody response to the HCoV‐OC43 S and N genes in these cases.

**TABLE 2:** The seropositivity rates (titers above detection threshold of 1:100) of anti‐SARS‐CoV‐2 S and N antibodies preseasonal and postseasonal coronavirus infection, compared with uninfected controls.

Infecting strain	*N*	SARS‐CoV‐2 S				SARS‐CoV‐2 N			
		Preinfection T0, *n* (%)	GMT (95% CI)	Postinfection T1, *n* (%)	GMT (95% CI)	Preinfection T0, *n* (%)	GMT (95% CI)	Postinfection T1, *n* (%)	GMT (95% CI)
HCoV‐229E	7	1 (14.3)	55 (43–70)	1 (14.3)	61 (38–99)	1 (14.3)	61 (38–99)	1 (14.3)	61 (38–99)
HCoV‐NL63	17	3 (17.6)	64 (47–87)	1 (5.9)	57 (44–73)	4 (23.5)	123 (51–296)	4 (23.5)	88 (46–171)
HCoV‐OC43	24	4 (16.7)	73 (46–114)	10 (41.7)	103 (60–177)	4 (16.7)	92 (50–169)	7 (29.1)	112 (61–206)
HCoV‐HKU1	14	—	—	1 (7.1)	55 (45–68)	1 (7.1)	53 (47–58)	2 (14.3)	58 (46–73)
Overall	62	8 (12.9)	—	13 (21.0)	—	10 (16.1)	—	14 (22.5)	—
HCoV PCR(−)	10	1 (10.0)	71 (32–155)	3 (30.0)	93 (40–220)	1 (10.0)	54 (46–63)	1 (10.0)	62 (38–99)

Among those with cross‐reactive antibody titers to SARS‐CoV‐2 and available serum samples at multiple timepoints, four individuals exhibited detectable anti‐S IgG titers at only a single timepoint, while four others showed transient anti‐N IgG responses. However, in most cases, cross‐reactive IgG antibodies targeting either the S or N protein persisted across more than two timepoints, indicating that the cross‐reactive antibody response was not transient.

**FIGURE 2:**
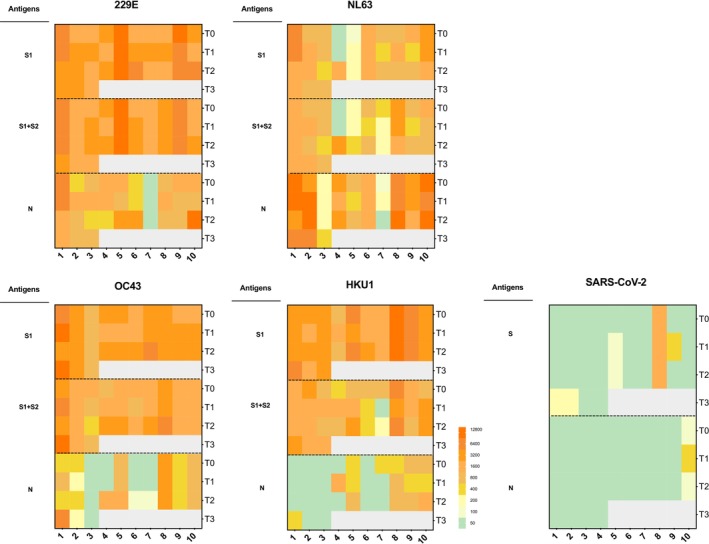
IgG antibody profile of HCoV‐negative individuals (*N* = 10) against seasonal coronavirus and SARS‐CoV‐2 antigens before and after self‐reported respiratory illness. Each panel included all 10 HCoV negative participants and showed IgG response to antigens of one particular HCoV. T0 represents the baseline, while T1 to T4 represent the first to fourth follow‐up after infection, respectively.

Additionally, we tested a control group comprising 10 participants who reported ARI but were PCR‐negative for all HCoVs during screening. Notably, one of these PCR‐negative controls (1/10) also showed an eight‐fold increase to SARS‐CoV‐2 spike (S) after ARI and one showed a four‐fold increase to nucleocapsid (N) proteins without concurrent HCoV seroconversion (Figure 2). This might be due to nonspecific immune responses to SARS‐CoV‐2 antigens or assay interference.

Taken together, these results indicate that very few older adults possessed cross‐reactive pre‐existing SARS‐CoV‐2 antibodies prior to its emergence and that a seasonal coronavirus infection only induced marginal cross‐reactive antibody responses to SARS‐CoV‐2 S and N antigens.

## 4 Discussion

Early during the emergence of COVID‐19, it was reported that older adults experienced higher rates of severe outcomes of SARS‐CoV‐2 infections compared with children, including higher hospitalization and mortality rates [11]. Follow‐up reports that pre‐existing SARS‐CoV‐2‐reactive CD4^+^ T cell responses were found to mirror the frequency and functional quality of OC43‐specific memory CD4^+^ T cells in childhood. This cross‐reactive T cell response, which declines with age, suggested a potential role for pre‐existing protective immunity, which may contribute to the distinct susceptibility observed in older adults and children [12].

In this study, we assessed whether seasonal coronavirus infection could elicit any cross‐reactive IgG antibody response to SARS‐CoV‐2 in a prepandemic cohort of older adults in Jiangsu, China. Here, our results suggested that in our prepandemic cohort most individuals had pre‐existing antibody titers against all seasonal HCoV viruses, but only 13%–16% of individuals had detectable pre‐existing antibodies against either SARS‐CoV‐2 S or N protein at low titers. We also found increased SARS‐CoV‐2 antibodies after seasonal coronavirus infections, although only a few OC43‐infected individuals showed seroconversion to SARS‐CoV‐2 antigens, while the frequencies were much lower and indistinguishable from background (HCoV PCR negative cases) for the other seasonal HCoVs. This suggests that SARS‐CoV‐2 cross‐reactive antibody responses rarely occurred and were likely only associated with HCoV‐OC43, the seasonal betacoronavirus with the highest sequence identity to SARS‐CoV‐2 [1]. It will be interesting to evaluate whether a recent HCoV‐OC43 infection will provide cross‐protection against SARS‐CoV‐2 infection, as it has been suggested that a high level of HCoV‐OC43 anti‐N antibodies reduced SARS‐CoV‐2 infection risk [13]. It also remains to be confirmed whether there is minimal cross‐reactivity between SARS‐CoV‐2 and HCoV‐HKU1, also a betacoronavirus.

There are several limitations in our study. First, our study population consisted of older adults, and therefore, the generalizability of our findings to younger age groups may be limited. Second, previous studies showed that S2 contributes more prominently to cross‐reactivity [14]. Here, we measured antibodies against S, which may not fully capture the specific contribution of anti‐S2 antibodies. Additionally, due to the requirement of BSL‐3‐level containment, we were unable to assess the neutralization ability in the participants with elevated serological reactivity to SARS‐CoV‐2. Finally, due to the variable infection timepoints, we had limited postinfection T3 and T4 samples from the participants who were infected later in the study, which restricted our analysis of long‐term antibody kinetics and durability.

In summary, although reported elsewhere that SARS‐CoV‐2 infections may boost pre‐existing antibodies targeting HCoV [15], our findings suggest that HCoV infections do not elicit significant cross‐reactive antibodies against SARS‐CoV‐2, even in older adult populations with prior immunological experience. These data therefore contribute to the understanding of coronavirus antigenic cross‐reactivity and have implications for the development of pan‐coronavirus vaccines.

## Author Contributions

**Cheng Xiao:** methodology, investigation, formal analysis, writing – original draft. **Nancy H.L. Leung:** methodology, investigation, validation, project administration, resources, writing – review and editing. **Shiman Ling:** methodology, investigation. **Xia Lin:** methodology, investigation, writing – review and editing. **Yuyun Chen:** project administration, resources. **Yanmin Xie:** project administration, resources. **Malik Peiris:** supervision, resources. **Mark Zanin:** formal analysis, supervision, project administration, writing – review and editing. **Benjamin Cowling:** investigation, supervision, funding acquisition, project administration, resources, writing – review and editing. **Sook‐San Wong:** investigation, validation, formal analysis, supervision, funding acquisition, project administration, writing – review and editing.

## Funding

This study was supported by the Theme‐based Research Scheme (Project No. T11‐705/21‐N) of the Research Grants Council of the Hong Kong SAR Government. CARES was supported by the US Centers for Disease Control and Prevention (Cooperative Agreement Number IP001064‐01).

## Ethics Statement

The study protocol received ethical approval from the Institutional Review Board of the University of Hong Kong (Ref: UW15 404), and the Ethics Committee of Jiangsu Provincial Center for Disease Prevention and Control (Ref: JSJK2015‐B013‐02).

## Conflicts of Interest

Benjamin Cowling has consulted for AstraZeneca, Fosun Pharma, GlaxoSmithKline, Haleon, Moderna, Novavax, Pfizer, Roche, Sanofi Pasteur, and Seqirus. Sook‐San Wong has received speakers' honorarium from Sanofi Pasteur. All other authors report no potential conflicts of interest.

## Data Availability

Data are available upon reasonable request from the authors.

## References

[1] N. Kaur , R. Singh , Z. Dar , R. K. Bijarnia , N. Dhingra , and T. Kaur , “Genetic Comparison Among Various Coronavirus Strains for the Identification of Potential Vaccine Targets of SARS‐CoV2,” Infection, Genetics and Evolution 89 (2021): 104490. 10.1016/j.meegid.2020.104490 PMC7395230 32745811

[2] K. W. Ng , N. Faulkner , G. H. Cornish , et al., “Preexisting and De Novo Humoral Immunity to SARS‐CoV‐2 in Humans,” Science 370 (2020): 1339–1343. 33159009 10.1126/science.abe1107 PMC7857411

[3] C. Y. Lin , J. Wolf , D. C. Brice , et al., “Pre‐Existing Humoral Immunity to Human Common Cold Coronaviruses Negatively Impacts the Protective SARS‐CoV‐2 Antibody Response,” Cell Host & Microbe 30 (2022): 83–96. 34965382 10.1016/j.chom.2021.12.005 PMC8648673

[4] M. Sagar , K. Reifler , M. Rossi , et al., “Recent Endemic Coronavirus Infection Is Associated With Less‐Severe COVID‐19,” Journal of Clinical Investigation 131 (2021): e143380. 32997649 10.1172/JCI143380 PMC7773342

[5] A. L. McNaughton , R. S. Paton , M. Edmans , et al., “Fatal COVID‐19 Outcomes Are Associated With An Antibody Response Targeting Epitopes Shared With Endemic Coronaviruses,” JCI Insight 7 (2022): e156372. 35608920 10.1172/jci.insight.156372 PMC9310533

[6] G. Song , W. T. He , S. Callaghan , et al., “Cross‐Reactive Serum and Memory B‐Cell Responses to Spike Protein in SARS‐CoV‐2 and Endemic Coronavirus Infection,” Nature Communications 12 (2021): 2938. 10.1038/s41467-021-23074-3 PMC8134462 34011939

[7] S. Nickbakhsh , A. Ho , D. F. P. Marques , J. McMenamin , R. N. Gunson , and P. R. Murcia , “Estimating Global Epidemiology of Low‐Pathogenic Human Coronaviruses in Relation to the COVID‐19 Context Reply,” Journal of Infectious Diseases 222 (2020): 696–698. 32497175 10.1093/infdis/jiaa321 PMC7377292

[8] B. J. Cowling , C. Xu , F. Tang , et al., “Cohort Profile: The China Ageing REespiratory Infections Study (CARES), a Prospective Cohort Study in Older Adults in Eastern China,” BMJ Open 7 (2017): e017503. 10.1136/bmjopen-2017-017503 PMC5695487 29092901

[9] N. H. Leung , H. Zhang , J. Zhang , et al., Incidence, Symptoms and Medical Care for Influenza Virus and Respiratory Syncytial Virus Illnesses among Older Adults in Eastern China: Findings from the China Ageing Respiratory Infections Study (CARES), 2015–2017. medRxiv, 2024.2007.2003.24309873 (2024).

[10] P. H. Niu , J. Shen , N. Zhu , R. J. Lu , and W. J. Tan , “Two‐Tube Multiplex Real‐Time Reverse Transcription PCR to Detect Six Human Coronaviruses,” Virologica Sinica 31 (2016): 85–88. 26826078 10.1007/s12250-015-3653-9 PMC7091384

[11] P. Zimmermann and N. Curtis , “Why Is COVID‐19 Less Severe in Children? A Review of the Proposed Mechanisms Underlying the Age‐Related Difference in Severity of SARS‐CoV‐2 Infections,” Archives of Disease in Childhood 106 (2021): 429–439. 33262177 10.1136/archdischild-2020-320338

[12] M. Humbert , A. Olofsson , D. Wullimann , et al., “Functional SARS‐CoV‐2 Cross‐Reactive CD4^+^ T Cells Established in Early Childhood Decline With Age,” Proceedings of the National Academy of Sciences of the United States of America 120 (2023): e2220320120. 36917669 10.1073/pnas.2220320120 PMC10041119

[13] A. H. A. Lavell , J. J. Sikkens , A. W. D. Edridge , et al., “Recent Infection With HCoV‐OC43 May Be Associated With Protection Against SARS‐CoV‐2 Infection,” Iscience 25 (2022): 105105. 36101832 10.1016/j.isci.2022.105105 PMC9458542

[14] E. Shrock , E. Fujimura , T. Kula , et al., “Viral Epitope Profiling of COVID‐19 Patients Reveals Cross‐Reactivity and Correlates of Severity,” Science 370 (2020): eabd4250. 32994364 10.1126/science.abd4250 PMC7857405

[15] E. M. Anderson , E. C. Goodwin , A. Verma , et al., “Seasonal Human Coronavirus Antibodies Are Boosted Upon SARS‐CoV‐2 Infection but Not Associated With Protection,” Cell 184 (2021): 1858–1864. 33631096 10.1016/j.cell.2021.02.010 PMC7871851

